# Near-Infrared Plasmonic Assemblies of Gold Nanoparticles with Multimodal Function for Targeted Cancer Theragnosis

**DOI:** 10.1038/s41598-017-17714-2

**Published:** 2017-12-11

**Authors:** Seong-Eun Kim, Bo-Ram Lee, Hohyeon Lee, Sung Duk Jo, Hyuncheol Kim, You-Yeon Won, Jeewon Lee

**Affiliations:** 1 Department of Chemical and Biological Engineering, Korea University, Seoul, 02841 Republic of Korea; 2 School of Chemical Engineering, Purdue University Center for Cancer Research, Purdue University, West Lafayette, IN 47906 USA; 3 Center for Theragnosis, Korea Institute of Science and Technology, Seoul, 02792 Republic of Korea; 4 Department of Chemical and Biomolecular Engineering, Sogang University, Seoul, 04107 Republic of Korea

## Abstract

Here we report a novel assembly structure of near-infrared plasmonic gold nanoparticles (AuNPs), possessing both photoacoustic (PA) and photothermal (PT) properties. The template for the plasmonic AuNP assembly is a bioconjugate between short double-strand DNA (sh-dsDNA) and human methyl binding domain protein 1 (MBD1). MBD1 binds to methylated cytosine-guanine dinucleotides (mCGs) within the sequence of sh-dsDNA. Hexahistidine peptides on the engineered MBD1 function as a nucleation site for AuNP synthesis, allowing the construction of hybrid conjugates, sh-dsDNA-MBD1-AuNPs (named DMAs). By varying the length of sh-dsDNA backbone and the spacer between two adjacent mCGs, we synthesized three different DMAs (DMA_5mCG, DMA_9mCG, and DMA_21mCG), among which DMA_21mCG exhibited a comparable photothermal and surprisingly a higher photoacoustic signals, compared to a plasmonic gold nanorod. Further, epidermal growth factor receptor I (EGFR)-binding peptides are genetically attached to the MBD1 of DMA_21mCG, enabling its efficient endocytosis into EGFR-overexpressing cancer cells. Notably, the denaturation of MBD1 disassembled the DMA and accordingly released the individual small AuNPs (<5 nm) that can be easily cleared from the body through renal excretion without causing accumulation/toxicity problems. This DMA-based novel approach offers a promising platform for targeted cancer theragnosis based on simultaneous PA imaging and PT therapy.

## Introduction

AuNPs have attracted much attention because synthetic methods are well developed, they exhibit unique and tunable optical (localized surface plasmon resonance (LSPR)) properties, and they are generally considered biocompatible, making them an attractive material for biomedical applications such as cancer therapy and imaging^1–4^. In particular, AuNPs can absorb light and transform it into heat, resulting in both a localized temperature increase and an acoustic wave generation. Thus AuNPs are considered as a promising dual functional agent for photothermal (PT) therapy and photoacoustic (PA) imaging of cancer^5–7^ and thus provide highly useful function for cancer theragnosis that simultaneously enables cancer diagnosis and therapy and monitoring of the responses to therapy. The cancer theragnosis can significantly reduce risks and cost in cancer treatment and improve cancer management, and is therefore highly attractive to both clinicians and patients^8–10^.

One problem to hamper the clinical application of AuNPs, however, is the accumulation of AuNPs inside the body, which causes nanotoxicity^11,12^. For example, the AuNPs accumulated in liver cause severe damages such as acute inflammation, tissue apoptosis, and abnormal increase of kupffer cells^13–15^. To avoid this problem, AuNPs must be sufficiently small, because small-sized AuNPs (<8 nm in diameter) that can pass through glomerular filtration are effectively cleared from the body through renal excretion, and thus result in less accumulation in body tissues^16–18^. Additionally, smaller AuNPs (<10 nm) typically show higher photothermal efficiencies, i.e. more efficiently transduce light to heat than larger AuNPs, because smaller AuNPs produce less surface light scattering. According to Mie theory that simulates the photothermal properties of spherical nanoparticles, absorption and scattering properties depend on size of the particles. As particle size increases, spherical particles gradually start to produce more surface light scattering^19^. Another important requirement for *in vivo* theragnostic AuNPs is that they should be able to absorb near-infrared (NIR) light in the wavelength range of 600 to 1,000 nm, which has sufficiently long tissue penetration depth appropriate for *in vivo* deep tissue imaging and therapy^20,21^. Although non-spherical gold nanoparticles such as gold nanorods (AuNRs) or gold nanoshells (AuNSs) are more capable of absorbing the NIR light, it is difficult to synthesize AuNRs or AuNSs that are sufficiently small to be cleared from the body through renal excretion^22,23^. Small spherical AuNPs with a diameter of about 5 nm have a maximum absorption peak at around 520 nm^24^ and are therefore not suitable for *in vivo* deep tissue application. A possible solution is to develop a rod-shaped assembly of small spherical AuNPs that can exhibit efficient plasmon absorption at NIR wavelengths due to plasmon coupling between individual AuNPs^25,26^.

In addition, targeted delivery of AuNPs to cancer cells has been another major issue for PT therapy. Higher targeting efficiency reduces the amount of therapeutically ineffective AuNPs that are delivered to the distant region from cancer and hence enhances the performance of PT cancer therapy^27^. To make AuNPs have a cancer-targeting capability, the surface of AuNPs has been chemically conjugated with cancer cell-binding biomolecules (bio-ligands such as peptides or antibodies having affinity for cancer cells)^28,29^. However, the chemical conjugation is generally performed in a random fashion, and therefore the critical factors affecting cancer-targeting function of AuNPs (i.e. surface density, orientation, and activity of bio-ligands attached on AuNPs) are not well-controllable, which still remains problematic. In this study, these problems was resolved by genetic conjugation that enables highly uniform conjugation of correctly oriented bio-ligands without conformational alteration to protein template used for ordered assembly of AuNPs^30^.

Reportedly, self-assemblies of biomacromolecules such as DNA and proteins have provided structural guides for producing ordered metal nanocrystal/nanowire arrays^31,32^: complementary DNA hybridization or avidin-biotin binding mechanism has mediated the construction of a structural template that AuNP superstructures are formed on. This method is typically based on thiol-linked conjugation between the template and small AuNPs (1 to 5 nm) that are formerly synthesized through the reduction of gold salts in the presence of thiol capping ligands, thus leaving the capping ligands on the AuNP surface, which may interfere with further surface modification and/or functionalization of the AuNPs^33,34^. In this study, we demonstrate a new method of AuNP assembly using a DNA-protein conjugate as a template for on-site synthesis of AuNPs. The DNA-protein conjugate template is prepared by the specific binding between human MBD1 and mCGs on sh-dsDNA, followed by AuNP synthesis on MBD1. The polyhistidine peptide on MBD1 serves as a nucleation site for AuNP synthesis. This procedure does not require the use of thiol capping ligands, and the size of AuNPs and the aspect ratio of their assembled structure are easily controllable. Notably, the sh-dsDNA-MBD1-AuNP assemblies show light absorption at NIR wavelengths, efficient photothermal and photoacoustic properties, and excellent cancer cell targeting capability. Furthermore, they are reversibly disassembled to small AuNPs that can be effectively cleared through renal excretion. The results of this study demonstrate that DMAs hold a promising potential as a platform agent for biocompatible and targeted cancer theragnosis.

## Result

### Synthesis and assembly of small AuNPs using DNA-protein conjugate as a template

**Figure 1:**
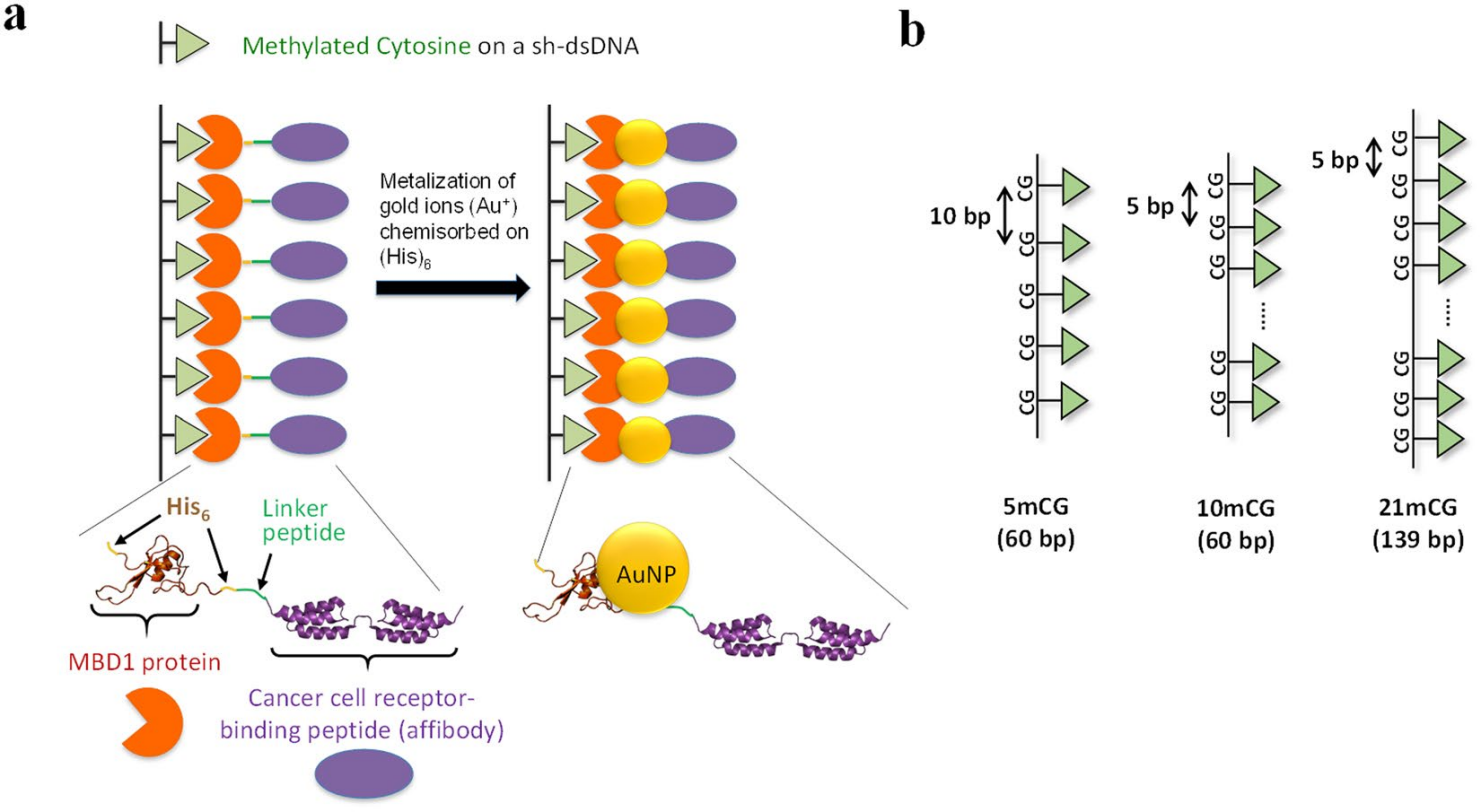
Schematic illustration of (**a**) DMA consisting of sh-dsDNA, MBD1, and AuNPs and (**b**) three sh-dsDNA backbones containing 5, 9, and 21 methylated cytosine-guanine (mCG) dinucleotides: 5 mCG, 9 mCG, and 21 mCG.

As shown in Fig. 1a, an MBD1 variant that is genetically engineered to have N- and C-terminal polyhistidine tags (H_6_) and C-terminal affibodies binds to the mCG sites of a sh-dsDNA backbone. Three different sh-dsDNAs having different sequence and length (named 5 mCG, 9 mCG, and 21 mCG) were used to examine the effects of mCG location and distance between mCG sites on the AuNP formation (Fig. 1b). The 5- and 9 mCG represent a 60-bp dsDNA backbone containing 5 mCGs with 10-bp spacer and 9 mCGs with 5-bp spacer, respectively. The 21 mCG represent a 139-bp dsDNA backbone containing 21 mCGs with 5-bp spacer. The polyhistidine tags on MBD1 were used as a site for the chemisorption of gold ions (Au^+^) and for the growth of AuNP^35^. That is, Au^+^ ions chemisorbed on H_6_ were metalized by addition of a reducing agent (NaBH_4_), leading to the formation of sh-dsDNA-MBD1-AuNPs (DMAs), as confirmed by energy dispersive X-ray (EDX) spectroscopy (Supplementary Fig. 1).

**Figure 2:**
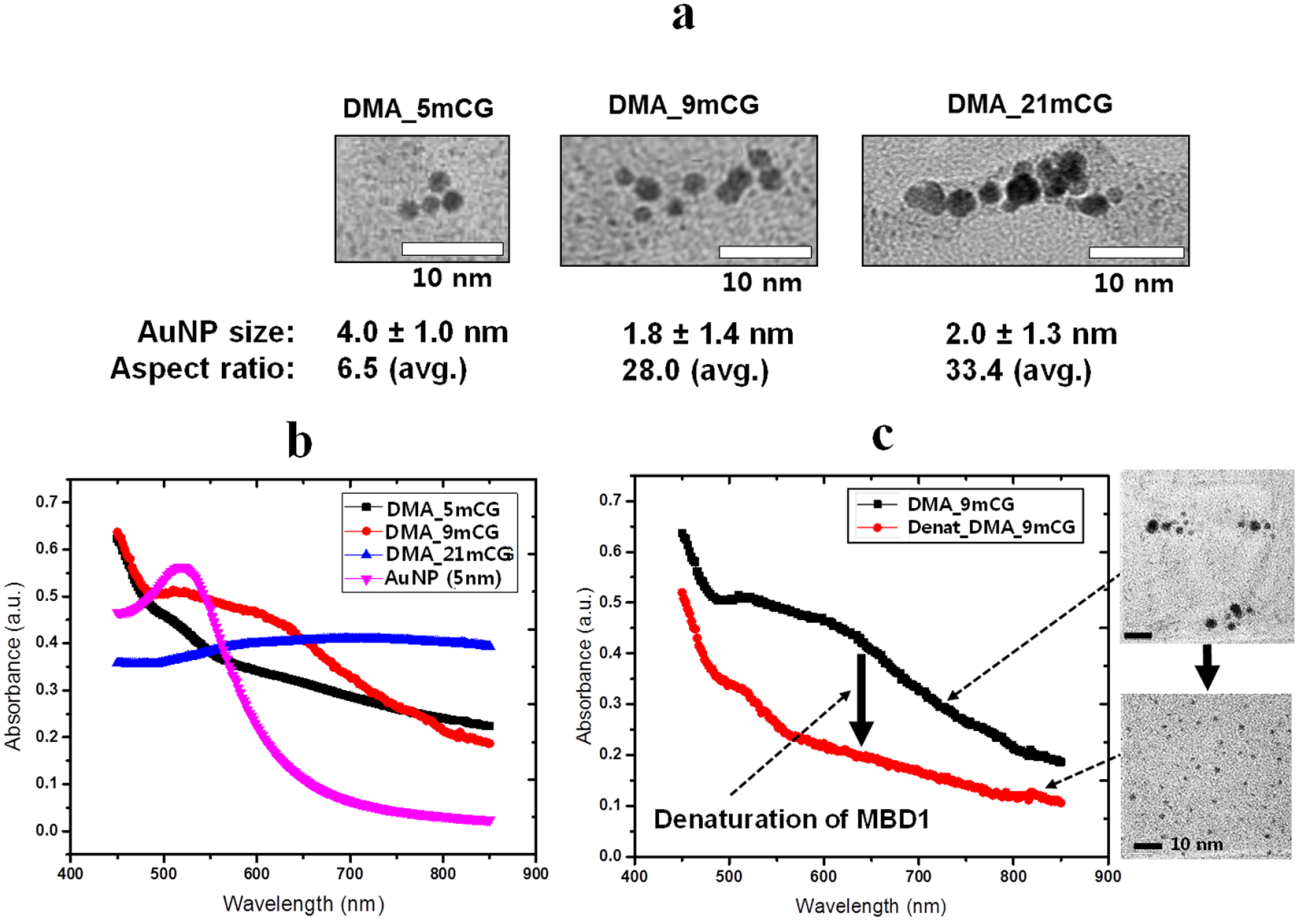
Morphological and optical characteristics of DMAs (DMA_5mCG, DMA_9mCG, and DMA_21mCG). (**a**) TEM images. (**b**) Light (UV/VIS/NIR) absorption spectra of three DMAs and AuNP (5 nm). (**c**) Light absorption spectra and TEM images of DMA_9mCG before and after MBD1 denaturation by Gdn-HCl (6 M for 10 min at room temperature).

From the analysis of transmission electron microscopy (TEM) (Fig. 2a), DMA synthesized using 5 mCG as a template (DMA_5mCG) consists of AuNPs with a diameter of ~4 nm, showing an aspect ratio of ~6.5, whereas 9- and 21 mCG-derived DMAs (DMA_9mCG and DMA_21mCG, respectively) show different aspect ratios (Table 1), ~28.0 and ~33.4, respectively but consist of the AuNPs with a similar size (~2 nm). It is speculated that the spacer length between mCGs on the DNA template significantly influences the size of AuNPs although the same amount of gold salt was used, which is because an AuNP growth is subject to be confined by adjacent AuNPs. Figure 2b shows that compared to standard AuNPs (5 nm), light absorption spectra of DMAs was significantly red-shifted, due probably to the confinement of multiple AuNPs within small DMA dimensions and plasmon coupling between adjacent AuNPs. It is notable that DMA_21mCG exhibited broader overall absorption spectra and a stronger NIR absorption at around 800 nm than the other two DMAs, which is because the magnitude of plasmon coupling increases as AuNP number per assembly increases.

**Table 1:** Physicochemical properties of DMAs used in this study.

DMA (backbone length, bp)	Zeta potential (mV)^a^	Spacer length between adjacent mCGs (nm)	Size of individual AuNPs on DMAs (nm)^b^	Surface-to-surface distance between adjacent AuNPs on DMAs (nm)^c^
DMA_5mCG (60 bp)	−10.6 ± 0.4	3.4 nm (10 bp)	4.0 ± 1.02	1.5 ± 1.25
DMA_9mCG (60 bp)	−12.1 ± 0.5	1.7 nm (5 bp)	1.8 ± 1.40	2.3 ± 1.01
DMA_21mCG (139 bp)	−12.3 ± 0.7	1.7 nm (5 bp)	2.0 ± 1.28	2.1 ± 2.12

^a^Measured using an Nano ZS Zetasizer.

^b,c^Estimated from TEM images.

When one of DMAs (i.e. DMA_9mCG) was treated with a protein denaturant (6 M guanidine hydrochloride, Gdn-HCl), it was completely disassembled to individual small AuNPs as shown in TEM images of Fig. 2c. This was also confirmed by the disappearance of the broad absorbance at NIR wavelengths (Fig. 2c), while the same Gdn-HCl treatment did not cause any change in the absorbance spectra of standard AuNPs (5 nm) (data not shown). The denatured MBD1 is subject to losing specific binding affinity for mCGs on sh-dsDNA backbone, leading to the disassembly of the DNA-MBD1 conjugate, and in turn releasing the individual AuNPs. Additionally, we confirmed that DMA_21mCG was also well disassembled when placed in a solution at 37 °C (i.e. *in vivo* physiological temperature) for a long period of time (6 h and overnight) (Supplementary Fig. 2). The effect of temperature on the disassembly of DMA_21mCG was further estimated by increasing the solution temperature to a range of 37 to 65 °C overnight, clearly demonstrating the complete disassembly of DMA_21mCG under the elevated temperature conditions (Supplementary Fig. 3). This strongly suggests that DMAs can be reversibly disassembled inside the body where spontaneous protein denaturation always happens.

### Photothermal and photoacoustic properties of DMAs

**Figure 3:**
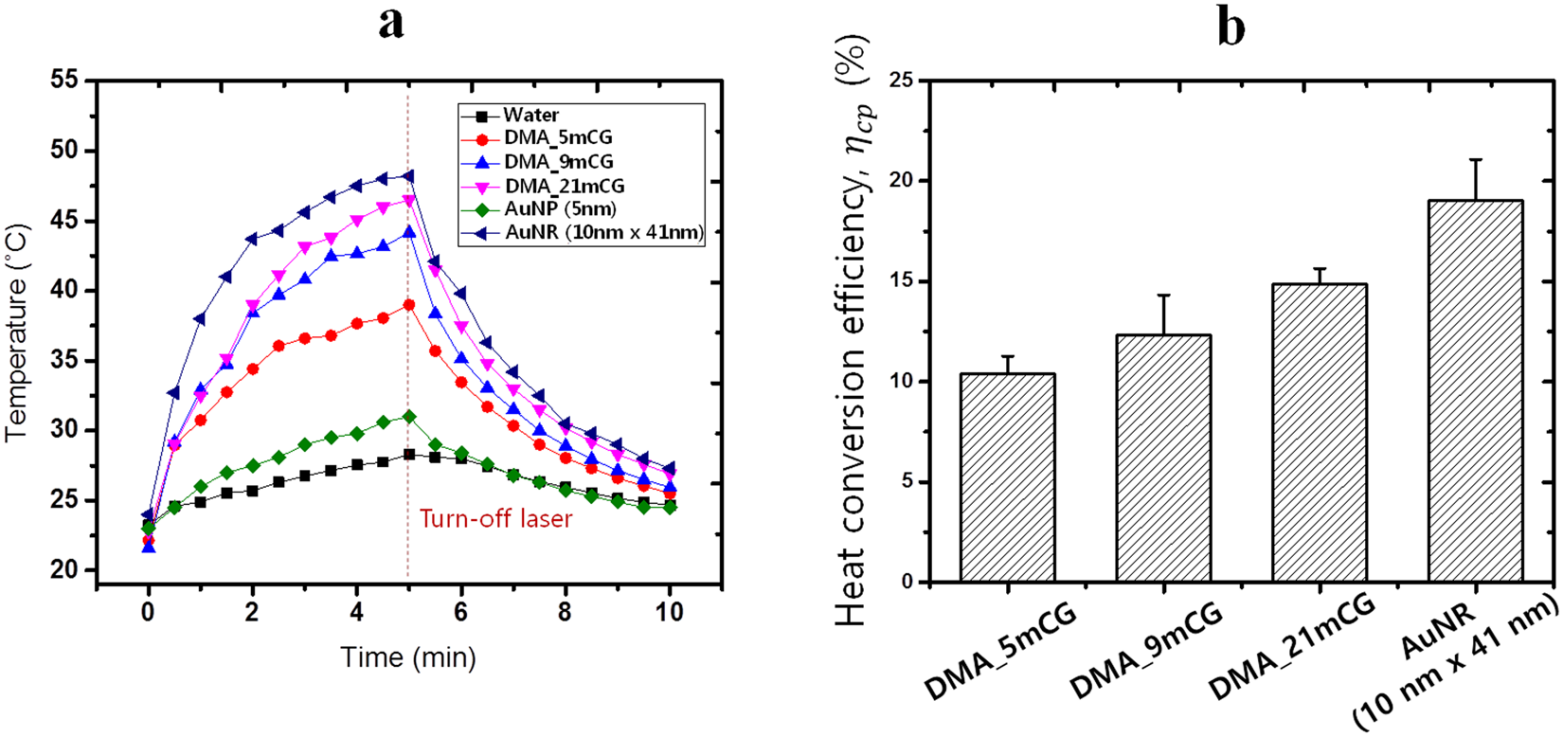
Photothermal activities of DMAs. (**a**) Time-course change of temperature of aqueous DMA/AuNP/AuNR solutions under continuous NIR laser irradiation (808 nm, 2 W/cm^2^, 5 min), followed by no irradiation (5 min). Blank water was used as a control. (**b**) Photothermal (PT) conversion efficiencies of DMAs and AuNR.

The potential utility of DMAs as a photothermal agent was evaluated by measuring the temperature elevation of aqueous DMA suspensions (0.02 mg Au per ml solution) under NIR laser irradiation (808 nm, 2 W/cm^2^), as presented in Fig. 3. After 5 min of NIR irradiation, the temperature of the DMA_5mCG, DMA_9mCG and DMA_21mCG solutions increased to 39.0, 44.2 and 46.5 °C, respectively. When tested under the same condition, the temperature of solutions containing commercial AuNPs (5 nm) and AuNRs (10 nm × 41 nm) increased up to 31.0 and 48.2 °C, respectively, and the temperature increase of pure water was negligible. In photothermal therapy of cancer, the tumor tissue is typically heated to 41 to 47 °C^5^, and therefore DMA_9mCG and DMA_21mCG show a potential as a PT agent at the low concentration used (0.02 mg Au/ml). Also as shown in Fig. 3a, upon cessation of NIR irradiation the DMA solutions were cooled down to room temperature within 5 min. By fitting these time-course temperature profiles to a theoretical model (Eqs 1 to 4 in Experimental Methods)^36,37^, we estimated the PT conversion efficiencies (*η*_*pc*_) of the DMAs. As summarized in Fig. 3b, the *η*_*pc*_ values of DMA_21mCG, DMA_9mCG, and DMA_5mCG were 14.8%, 12.3%, and 10.4%, respectively, while *η*_*pc*_ of the commercial AuNR (10 nm × 41 nm) was 19.0%. This result is in agreement with the previous report that smaller AuNPs have higher photothermal conversion efficiencies than larger AuNPs^13^. Among DMAs, DMA_21mCG shows the highest heat generation efficiency due probably to its highest aspect ratio and correspondingly strongest plasmon coupling of AuNPs.

**Figure 4:**
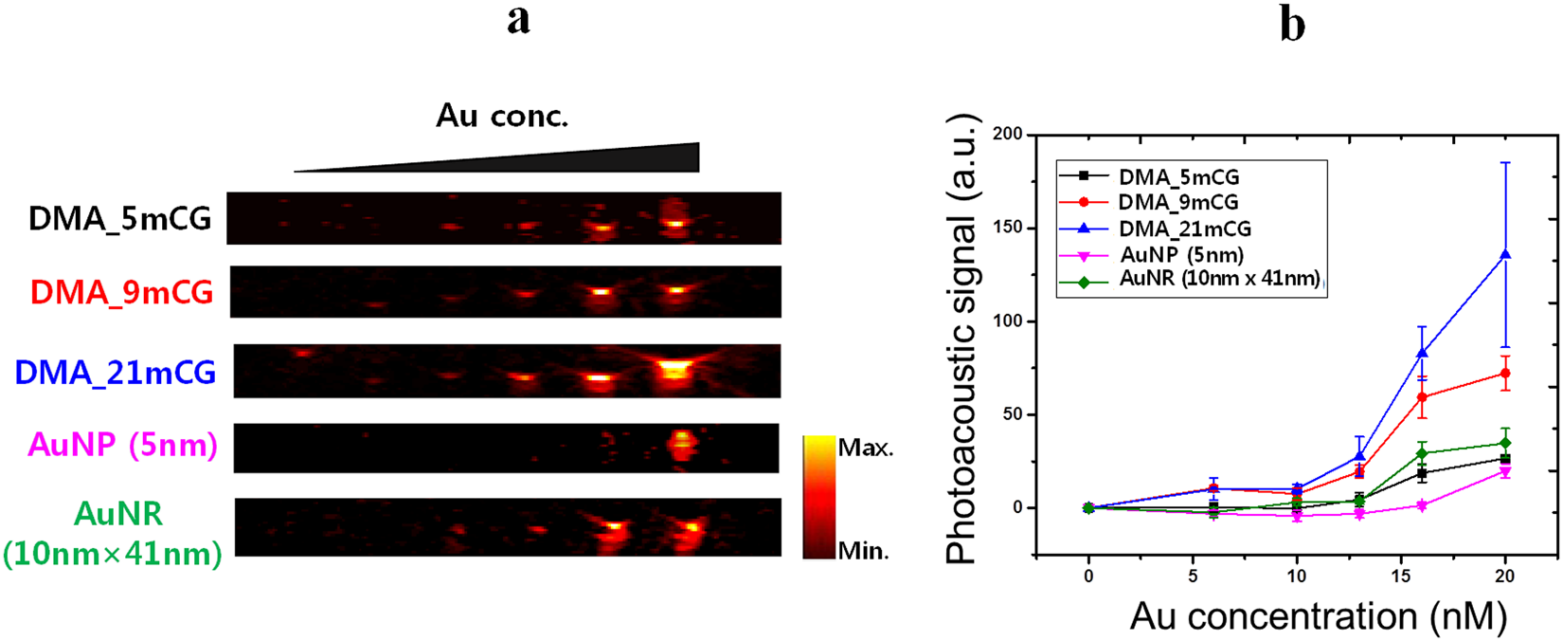
Photoacoustic activities of DMAs. (**a**) Photoacoustic (PA) images. (**b**) PA signal intensities of aqueous DMA/AuNP/AuNR solutions at various Au concentrations.

The PA properties of DMAs at various concentrations were estimated under NIR irradiation (808 nm wavelength). As presented in Fig. 4, the PA signal intensity monotonically increased with Au concentration, and DMA_21mCG showed the strongest PA signal, which is well explainable by PT efficiencies of DMAs because the PA signal is generated due to thermoelastic expansion (vibration) of AuNPs caused by locally heated media. It is worth noting that DMA_21mCG and DMA_9mCG generated stronger PA signal than AuNR (10 nm × 41 nm), even though the AuNR showed a higher PT efficiency than DMAs (Fig. 3). It is speculated that the close proximity between AuNPs on DMAs produced overlapping thermal fields and thus increased the thermal flux, resulting in an amplification of the PA signal^38^. Figures 3 and 4 indicate that DMA_21mCG has a strong potential as a cancer theragnostic agent based on PA imaging and PT therapy.

### Endocytic uptake of DMAs by *in vitro* cancer cells

**Figure 5:**
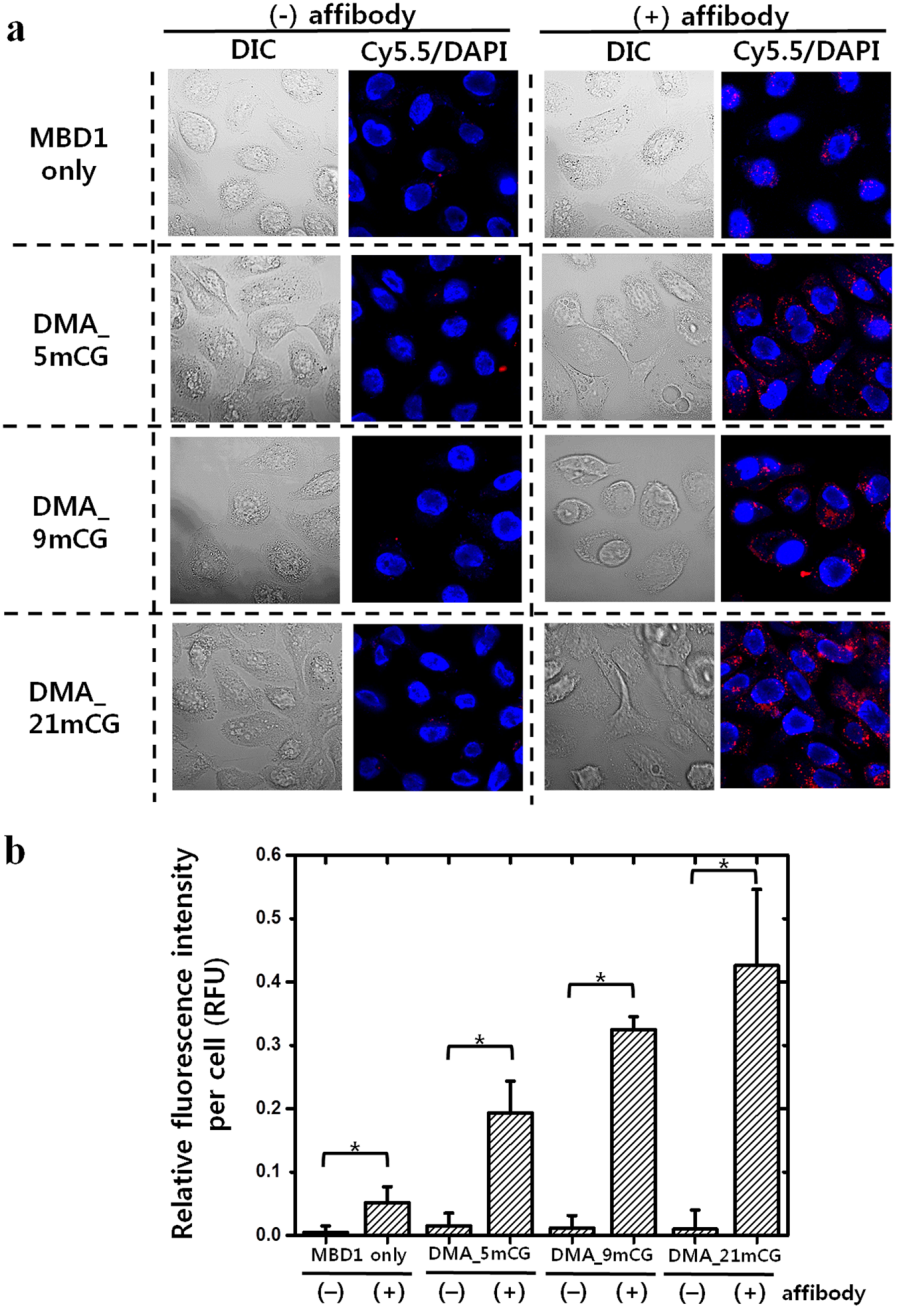
Cellular uptake of Cy5.5-labeled DMAs (aff+ and aff−) and MBD1 (aff+ and aff−) in EGFR-overexpressing A431 cancer cell cultures at 37 °C. (**a**) Confocal fluorescence microscopy images of A431 cells treated with Cy5.5-labeled MBD1 or DMAs with and without affibodies for 1 h. Nuclei were counterstained with DAPI (blue). (**b**) Fluorescence intensities from the images of (**a**). Error bars represent standard deviations (n ≥ 7). *p < 0.05.

As shown in Fig. 5a,b, the DMAs containing affibody (peptide ligand with high affinity for EGFR) (aff+) were effectively internalized by EGFR-expressing cancer (A431) cells, while the endocytic uptake of affibody-free DMAs by A431 cells was negligible. Notably, DMA_21mCG (aff+) that contains the highest number of mCG and MBD1 (aff+) exhibited the highest-level endocytic uptake by A431 cells among the three DMAs, and its uptake was almost 8-times higher than that of MBD1 (aff+) only (Fig. 5b), indicating that the cellular uptake of DMA increased as the number of MBD1 (aff+) per DMA (i.e. the number of mCG per DMA) increased. This result is in agreement with the previous finding that the multi-valent binding ligands significantly enhance the efficiency of binding to target^39^. Therefore, DMA-21mCG appears to be the best candidate for cancer theragnosis. Based on the previous literature^27,30^, the intracellular localization of DMA_21mCG can be determined from fluorescent cell images: punctate fluorescence patterns typically imply the confinement of fluorescent molecules within endosomal compartments such as lysosome, whereas smeared/diffuse fluorescent signals indicate the signaling molecules in cytosol. Figure 5 clearly shows the punctuate signals in tumor cells, suggesting that the intracellular DMAs are located in endosomal compartments.

### Cancer cell ablation using photothermal activity of DMAs

**Figure 6:**
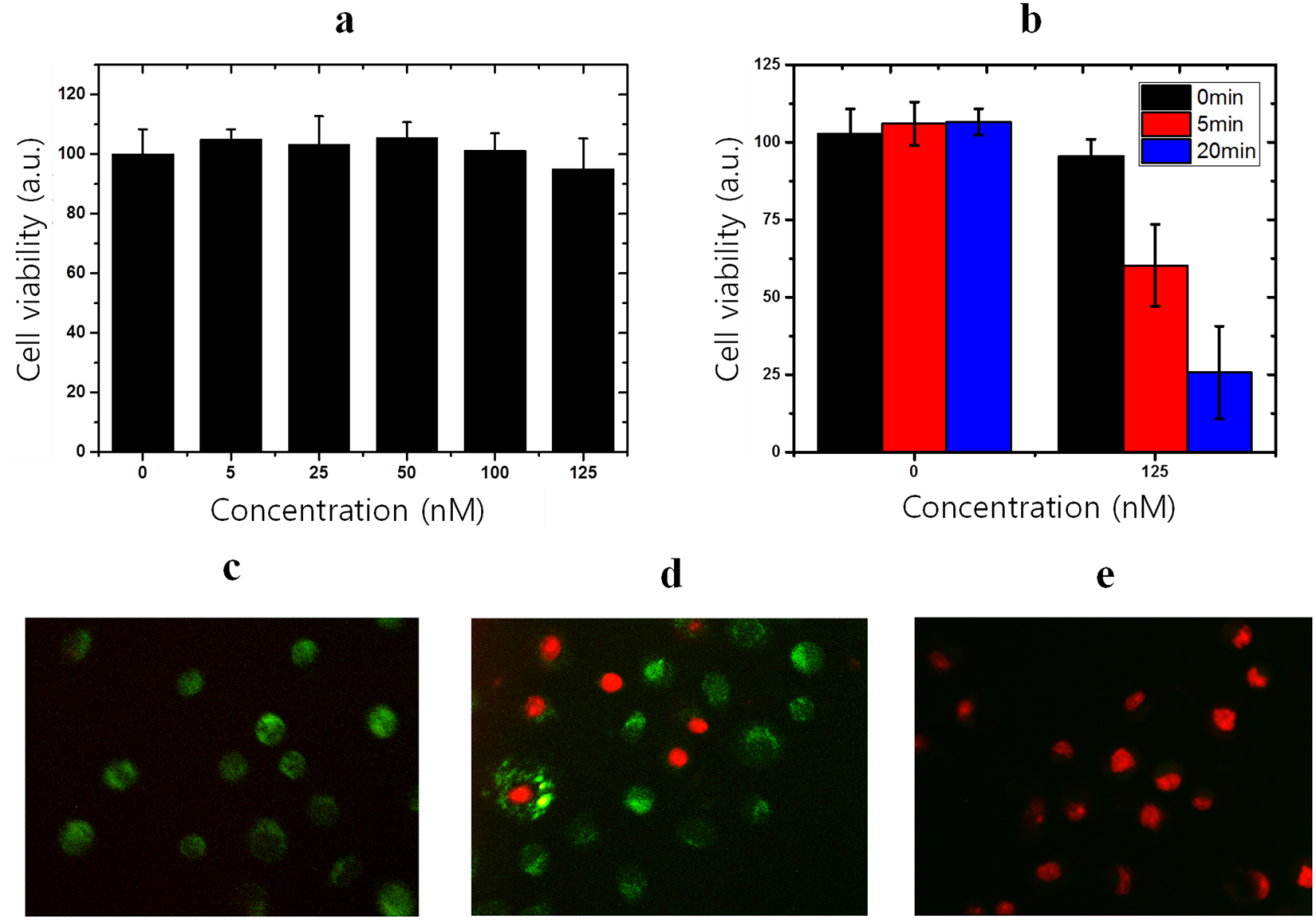
Cytotoxicity of and photothermal ablation of EGFR-overexpressing A431 cells by DMA_21mCG (aff+). (**a**) Cytotoxicity of DMA-21mCG in A431 cells treated at different concentrations for 24 h. (**b**) CCK-8 viability of A431 cells exposed to NIR irradiation (5 and 20 min) after treated by DMA_21mCG (aff+) (125 nM). Measurements were performed in triplicates. Error bars represent standard deviations. Fluorescence images of DMA_21mCG (125 nM)-treated A431 cells, double stained with Calcein AM and PI after an NIR laser irradiation (**c**) for 0-, (**d**) 5-, and (**e**) 20 min.

The cytotoxicity of DMA_21mCG in A431 cells was evaluated by the Cell Counting Kit-8 (CCK-8) method. As shown in Fig. 6a, when treated with DMA_21mCG (aff+) without NIR irradiation for 24 h, more than 95% of cells were viable at the entire range of concentration (up to 125 nM), indicating no cytotoxicity. Figure 6b shows that nearly 75% of the A431 cells was ablated at 2 h after the 20-min irradiation of NIR laser (808 nm, 2 W/cm^2^) to the cell cultures treated with DMA_21mCG (aff+) (125 nM), and the cancer cell death increased as the duration of NIR irradiation increased, indicating the excellent performance of PT-based cancer cell treatment by DMA_21mCG (aff+). As shown in Fig. 6c to e, the fluorescence images taken after staining with Calcein AM and propidium iodide (PI) confirmed that a significant level of cancer cell death occurred after the NIR treatment in the presence of intracellular DMA-21mCG (aff+). These results suggest that DMA_21mCG (aff+) is a promising functional material for targeted cancer therapy.

## Discussion

Cancer theragnosis that simultaneously enables cancer diagnosis and therapy is a next mainstay of clinical cancer treatment. It has crucial advantages over traditional cancer diagnoses and therapies and is highly attractive to both clinicians and patients because it can significantly reduce risks and cost in cancer treatment and improve cancer management. The multi-functional agents with cancer targeting, imaging, and therapeutic capabilities are essential to the cancer theragnosis, and here we report a new type of cancer theragnostic agent based on photothermal and photoacoustic activies of AuNPs. The rod-shaped assemblies of small AuNPs (<5 nm) that exhibit the light absorption at a broad range of NIR wavelengths were developed using MBD1-bound sh-dsDNA as a structural template, where MBD1 protein is biologically conjugated with sh-dsDNA via specific binding between MBD1 and mCG within the DNA sequence. The MBD1 protein was genetically modified by inserting two different foreign peptides to its N- and C-terminus: 1) histidine-rich peptides (H_6_) to be used as a gold ion (Au^3+^) chemisorption and gold nucleation site for on-site synthesis of the small AuNPs and 2) cancer cell receptor-binding peptides (EGFR-specific affibodies) to enhance cancer cell-targeting efficiency. Among the resulting DNA-MBD1-AuNP assemblies (DMAs), DMA_21mCG with the highest aspect ratio of rod-shaped assembly of AuNPs showed the highest-level PT and PA activities and the best performance in targeting EGFR-expressing cancer cells, suggesting that DMA_21mCG holds a promising potential as a cancer theragnostic agent. Further, it is worth noting that under the particular conditions for denaturing MBD1, the DMAs are disassembled and release the small individual AuNPs that can easily pass through glomerular filtration in kidney. This suggests that inside the body where proteins are spontaneously denatured, the DMA structure would be eventually atomized into small individual AuNPs that can be cleared from the body through renal excretion, which hence would resolve *in vivo* accumulation-associated toxicity problems of AuNPs that have been hampering *in vivo* clinical application of AuNPs.

In summary, we first report the synthesis of rod-shaped assemblies of small AuNPs with multimodality function (PT and PA activities at a broad range of NIR wavelengths), which was performed using genetically modified MBD1-bound sh-dsDNA as a synthetic scaffold. The three different sequences of sh-dsDNA with different length (60, 139 bp), different number of mCG (5, 9, 21), and different spacer length between adjacent mCGs (5, 10 bp) were used as a DNA template, where each mCG specifically binds to a single MBD1 protein. Gold ions (Au^3+^) are chemisorbed to histidine-rich peptide (H_6_) of engineered MBD1, immediately followed by reduction and nucleation to small AuNPs. The size of individual AuNPs and the aspect ratio of overall rod shape of assemblies were controlled through varying the length of both the spacer between adjacent mCGs and the entire sh-dsDNA backbone, resulting in three different assembly structures of AuNPs, i.e. DMA_5mCG, DMA_9mCG, and DMA_21mCG. Among the three DMAs, DMA_21mCG showed the highest PT and PA activities that are comparable to or much better than a commercial AuNR. Furthermore, DMA_21mCG comprising cancer cell receptor (EGFR)-binding affibody peptides (DMA_21mCG (aff+)) was successfully endocytosed by EGFR-expressing cancer cells (A431) without causing any cytotoxicity. The 20-min irradiation of NIR laser to A431 cells treated with DMA_21mCG (aff+) ablated about 75% of the initial cancer cells within 4 h. Notably, DMA_21mCG (aff+) was disassembled by denaturing MBD1, resulting in the release of the small individual AuNPs that can easily pass through glomerular filtration in kidney. Consequently, this novel approach to synthesize rod-shaped DMA assemblies enables the successful preparation of multimodality agent with cancer targeting, PA-based cancer imaging, and PT-based cancer therapeutic functions. DMAs could be cleared from the body through renal excretion without causing *in vivo* accumulation-associated toxicity problems. All these features make this novel DMA assemblies an attractive candidate as a cancer theragnostic agent.

## Methods

### Preparation of genetically engineered MBD1 and sh-dsDNA backbones

Through polymerase chain reactions (PCRs) using appropriate primers, two gene clones were prepared from previously cloned expression vectors for the synthesis of two MBD1 protein derivatives containing two hexahistidine tags: *NH*_2_-*Nde*I-H_6_-MBD1 (MAEDWLDSPALGPGWKRREVFRKSGATAGRSDTYYQSPTGDRIRSKVELTRYLGPAGDLTLFDFKQGIL)-H_6_-*Xho*I-*COOH* and *NH*_2_-*Nde*I-H_6_-MBD1-*Xho*I-H_6_-L(G_3_SG_3_TG_3_S G_3_)-(affibody)_2_-*Cla*I-*COOH*. These genes were sequentially ligated into the pT7-7 expression vector to construct the following vectors encoding the two engineered MBD1 proteins above: pT7-H6-MBD1-H6 and pT7-H6-MBD1-H6-Affi (Supplementary Fig. 4). *E. coli* Rosetta^TM^ (DE3) (Novagen, Darmstadt, Germany) was transformed with each expression vector above, and ampicillin-resistant transformants were selected. The recombinant *E. coli* cells were grown in Luria-Bertani media at 37 °C, and the recombinant gene expression was induced by adding Isopropyl-β-D-thiogalactoside (IPTG, 1 mM) to the growing culture when the culture turbidity (optical density at 600 nm, OD_600_) reached 0.5 to 0.7, followed by further cultivation for 6 h at 37 °C. The synthesized recombinant MBD1 proteins were purified using a Ni-NTA purification system (Qiagen, Hilden, Germany), as described in detail in our previous publication^40^. The purified MBD1 derivatives were analyzed by sodium dodecyle sulfate-polyacrylamide gel electrophoresis (SDS-PAGE) (18%) (Supplementary Fig. 4b).

Three different sh-dsDNA backbones were prepared by a commercial supplier (Xenotech, Daejeon, South Korea): two DNA backbones containing five CG and 9 CG dinucleotides, respectively, on 60-bp DNA fragment and a DNA backbone containing twenty one CG dinucleotides on 139-bp DNA fragment. The full sequences of the three sh-dsDNA backbones are presented in Table S1. The cytosine methylation of CG dinucleotides was performed using DNA methyltransferase (New England Biolabs, Massachusetts, USA), resulting in the synthesis of 5 mCG, 9 mCG, and 21 mCG. The degree of methylation was evaluated through analyzing the digestion pattern by *Hpa*II restriction enzyme as described in previous publication^40^ (also see Supplementary Fig. 5). In all methylated DNA products the degree of methylation was more than 99%.

### Synthesis and characterizations of DMAs

The sh-dsDNA-MBD1 conjugates were prepared as follows: a solution containing purified MBD1 protein (10 µM) with or without affibodies was directly mixed with a solution containing one of methylated DNA backbones (5 mCG, 9 mCG, or 21 mCG) at a molar ratio of sh-dsDNA to MBD1 of 1:200. The mixture was then incubated for 1 h at room temperature, followed by adding 1 mg of AuClP(CH_3_)_3_ (Sigma-Aldrich, St. Louis, MO, USA) to 200 µl of the mixture above (10 µM sh-dsDNA-MBD1 conjugate, 10 mM Tris-HCl, 1 mM ethylenediaminetetraacetic acid (EDTA), 1 mM dithiothreitol (DTT), pH 7.9). After stirring overnight at 20 °C, the mixture was centrifuged at 10,000 rpm for 15 min. The supernatant was mixed with 5 μl of 1 M NaBH_4_ (reducing agent), and the mixture was kept under stirring at 20 °C for 10 min to synthesize DMA. Among various types of reducing agents, NaBH_4_ was finally selected through preliminary studies to optimize the conditions for Au^3+^ reduction and DMA synthesis including the type and concentration of reducing agent, mixing and reduction time, *etc*.^41,42^.

The surface charge properties of DMAs (DMA_5mCG, DMA_9mCG, and DMA_21mCG) (10 nM DMA, 10 mM Tris-HCl, 1 mM EDTA, 1 mM DTT, pH 7.9) were characterized using a Nano ZS Zetasizer (Malvern Instruments, Worcestershire, UK) equipped with a 633 nm laser. The synthesized DMAs were examined by Transmission Electron Microscopy (TEM) and Energy Dispersive X-ray (EDX) spectroscopy using a CM-200 electron microscope (Philips, CA, USA) operating at an accelerating voltage of 200 kV. TEM and EDX spectroscopy specimens were prepared by dropping a 10 µL of 1 µM DMA suspension onto a carbon-coated copper grid. After waiting for 1 h (to allow the DMA to deposit on the grid surface), the TEM and EDX grid was washed twice with distilled water. For EDX spectroscopy analysis, the count number was plotted as a function of characteristic energy. UV/Vis absorption spectra for DMA samples were measured using a TECAN microplate reader (Infinite M200 Pro, TECAN, Männedorf, Switzerland).

### Photothermal conversion efficiencies (η_pc_) of DMAs

The photothermal properties of DMAs, gold nanorod (AuNR) (10 nm × 41 nm), and gold nanoparticle (AuNP) (5 nm) (Sigma-Aldrich, St. Louis, MO, U.S.A.) were characterized. The DMA, AuNR, and AuNP samples (20 µg Au/ml) were irradiated using an NIR laser (808 nm wavelength, 2 W/*cm* ^2^ laser power) for 5 min. The Au concentrations of DMA samples were determined by Inductively Coupled Plasma-Mass Spectrometry (ICP-MS) (Seoul Center, Korea Basic Science Institute (KBSI), Seoul, South Korea), and a correlation between Au and MBD1 concentrations of DMA were determined (Supplementary Fig. 6).

The phothothermal conversion efficiencies (η_pc_) of the DMA samples were estimated by comparing the measured cooling temperature profiles with the energy balance for the system^30,31^.

1 $${\eta }_{pc}=\frac{hS({T}_{max}-{T}_{surr})-{Q}_{in,surr}}{I(1-{10}^{A}\lambda )}$$

where *h*, *S*, *T*_*max*_, *T*_*surr*_, *Q*_*in,surr*_, and *I* are heat transfer coefficient, surface area of the container, steady-state maximum temperature, temperature of the surrounding, heat dissipation due to the absorptoin of the light by the medium (a value of 25.7 mW has been reported for pure water)^30,31^, and laser power in the units of mW, respectively. The value of *hS* can be estimated using the equation

2 $${\tau }_{s}=\frac{{m}_{w}{C}_{w}}{hS}$$

where τ_*s*_, *m*_*w*_, and *C*_*w*_ is system time constant, mass of the medium (water) (0.3 g), and heat capacity of water (4.2 J/g), respectively. As presented in Supplementary Fig. 7, the value of τ_*s*_ can be estimated by fitting measured cooling temperature profiles to the equations

3 $${\rm{t}}=-{\tau }_{s}\,\mathrm{ln}(\theta )$$

4 $$\theta =\frac{{T}_{surr}-T}{{T}_{surr}-{T}_{max}}$$

where t, *θ*, and *T* is cooling time, dimentionless driving force temperature, and temperature of the system at time t, respectively.

### Photoacoustic signal measurement

The PA properties of DMAs (DMA_21mCG, DMA_9mCG, DMA_5mCG), AuNP and AuNR were characterized as follows: Tygon tubes loaded with all samples to be tested were placed across at the center of a water bath and imaged using the procedure described in a previous publication^43^. An Nd:YAG laser system (Surelite III-10/Surelite OPO Plus, Continuum, Santa Clara, CA, USA) with an energy density of 6 mJ/cm^2^ was used as the excitation source. A linear array ultrasound (US) transducer with a frequency range of 5 to 14 MHz was used as a PA signal detector. To examine the effect of Au concentration on the PA signal intensity, 808 nm wavelength laser pulses were delivered under a constant irradiation power (6 mJ/cm^2^). The resulting PA signals were recorded using an US transducer. Photoacoustic signals were reconstructed into PA images by averaging 128 sequential frames, which gave a sufficient signal-to-noise ratio. After selecting a region of interest (ROI) in the reconstructed image, the total PA signal intensity from the ROI was quantified using MATLAB.

### *In vitro* cancer cell culture and fluorescence imaging

The cellular uptake efficiencies of MBD1 protein and DMAs were estimated in the culture of EGFR-overexpressing cancer (A431) cells. The A431 cells were cultivated using RPMI Medium 1640 (Gibco, Life technologies, Massachusetts, USA) containing fetal bovine serum (10%) and penicillin (100 U/ml), seeded in 35-mm coverslip bottom dishes at a density of 1 × 10^4^ cells/dish, and further cultivated for 24 h. After washing with phosphate buffer saline (PBS, pH 7.4), A431 cells were incubated in a serum-free medium containing Cy5.5-DMA (aff-) (50 nM), Cy5.5-DMA (aff+) (50 nM), or Cy5.5-MBD1 only (50 nM) for 2 h. After washed with PBS (pH 7.4), the Cy5.5-DMA- or Cy5.5-MBD1-treated cells were fixed in 4% paraformaldehyde and 0.1% glutaraldehyde for 10 min and then counterstained with 4′,6′-diamidino-2-phenylindole hydrochloride (DAPI). After washed once again with PBS, the cells in PBS were imaged using a Confocal Laser Scanning Microscope (LSM 700, Carl-Zeiss, Jena, Germany). Fluorescence measurement was performed using 630–680 nm excitation and 690–740 nm emission long pass filters. To quantify the fluorescence intesntity per cell, fluorescence images were analyzed using the ImageJ software as described in our previous publication^24^.

### Cytotoxicity of DMAs and photothermal ablation of cancer cells by DMAs

The cytotoxicity of DMA_21mCG in A431 cells was evaluated by Cell Counting Kit-8 (CCK-8). A431 cells were seeded in a 96-well microplate at a density of 1 × 10^4^ cells per well and cultivated for 24 h at 37°C in Roswell Park Memorial Institute (RPMI) Medium 1640 (Gibco, Life technologies, Massachusetts, USA) containing fetal bovine serum (10%) and penicillin (100 U/ml). Cells in different wells were treated with DMA_21mCG at different DMA concentrations (0, 5, 25, 50, 100 and 125 nM) and further incubated for 24 h. Finally, the DMA_21mCG-treated cells were stained using the CCK-8 kit for 2 h, and the absorbance of each well was measured at 450 nm in a microplate reader.

The photothermal effect of DMA_21mCG on the ablation of A431 cells was evaluated using the same CCK-8 method. A431 cells were seeded in a 96-well microplate (1 × 10^4^ cells/well) and cultivated for 24 h. Cells in different wells were treated with different concentrations of DMA_21mCG (aff+) (0 and 125 nM) and further incubated for 4 h. Then, the cells were irradiated by an NIR laser beam (808 nm, 2 W/cm^2^) for 0, 5, or 20 min. The CCK-8 reagent was added in each well, and the cells were incubated for 2 h, immediately followed by measuring the absorbance at 450 nm. The cell viability was determined as the ratio of the number of live cells in the DMA-treated cell culture to the number of live cells in non-DMA-treated control. Data were presented as means ± standard deviations for triplicate experiments. The cell viability was also estimated using Live/Dead Double Staining Kit (Sigma-Aldrich, St. Louis, MO, USA). Briefly, A431 cells were seeded in 35-mm coverslip bottom dishes (1 × 10^5^ cells per dish) and cultivated for 24 h. DMA_21mCG (aff+) (125 nM) was added to each coverslip, and the cells were incubated for 4 h. Afterwards, the cells were irradiated by an NIR laser (808 nm, 2 W/cm^2^) for 0, 5, or 20 min. The staining solution was prepared by adding 1 μl of Calcein acetoxylmethyl (Calcein AM) solution (1.5 mg/ml) and 2 μl of propidium iodide (PI) solution (1 mg/ml) to 2 ml PBS. This freshly prepared staining solution was added to each coverslip, and the cells were incubated for 15 min at room temperature. After the staining, the cells were imaged using a fluorescence microscope (Olympus IX81, Tokyo, Japan). Calcein AM (495 nm excitation, 515 nm emission) stains live cells (green), and PI (535 nm excitation, 617 nm emission) stains dead cells (red).

## Electronic supplementary material

supplementary dataset
