# Supplementary material for: Near-Infrared Plasmonic Assemblies of Gold Nanoparticles with Multimodal Function for Targeted Cancer Theragnosis
Source: Sci Rep. 2017 Dec 11;7:17327. doi: 10.1038/s41598-017-17714-2 (PMC5725556; doi:10.1038/s41598-017-17714-2)
Supplement: Supplementary file 1 — supplementary dataset [file 41598_2017_17714_MOESM1_ESM.doc]

Supporting Information

Near-Infrared Plasmonic Assemblies of Gold Nanoparticles with Multimodal Function for Targeted Cancer Theragnosis

Seong-Eun Kim^§^, Bo-Ram Lee^§^, Hohyeon Lee, Sung Duk Jo, Hyuncheol Kim, You-Yeon Won, and Jeewon Lee*

^§^ These authors contributed equally to this work

S.-E. Kim, B.-R. Lee, Prof. J. Lee

Department of Chemical and Biological Engineering,

Korea University,

Seoul 02841, Republic of Korea

*E-mail: leejw@korea.ac.kr

S.-E. Kim, Prof. Y.-Y. Won

School of Chemical Engineering, and Purdue University Center for Cancer Research,

Purdue University,

West Lafayette, IN 47906, USA

Dr. S. D. Jo, Prof. Y.-Y. Won

Center for Theragnosis, Korea Institute of Science and Technology, Seoul 02792, Republic of Korea

H. Lee, Prof. H. Kim

Department of Chemical and Biomolecular Engineering, Sogang University, Seoul 04107, Republic of Korea

**Revised manuscript (*SREP-17-38851)* submitted to**

***Scientific reports***

**Supplementary Figures**

**Figure S1.**

**
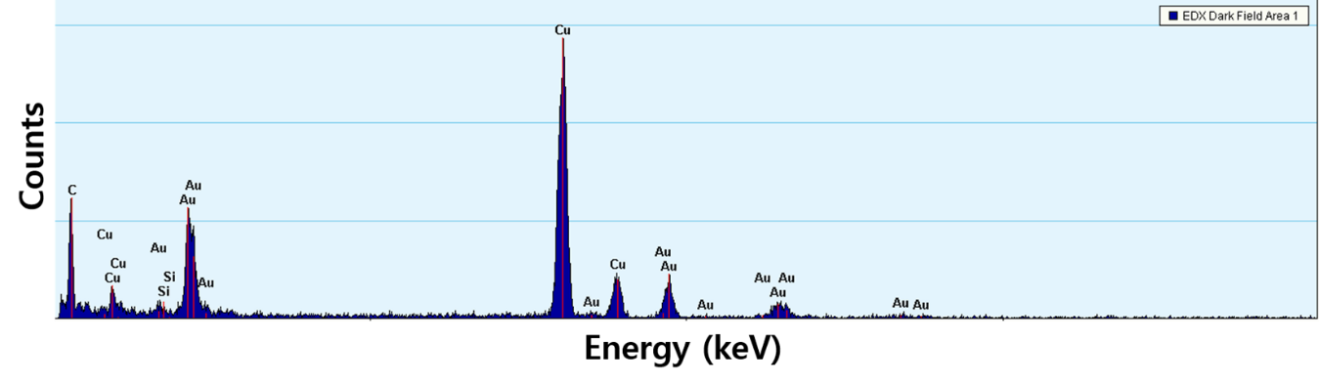
**

**Figure S1.** EDX count number vs. energy plot for DMA-21mCG.

**Figure S2.**

**Figure S2.** TEM images showing the disassembly of DMA_21mCGs that were placed for 6 h and overnight at 37 ^o^C.

**Figure S3.**

**Figure S3.** TEM images for showing the disassembly of DMA_21mCGs that were placed overnight at various temperatures.

**Figure S4.**

**
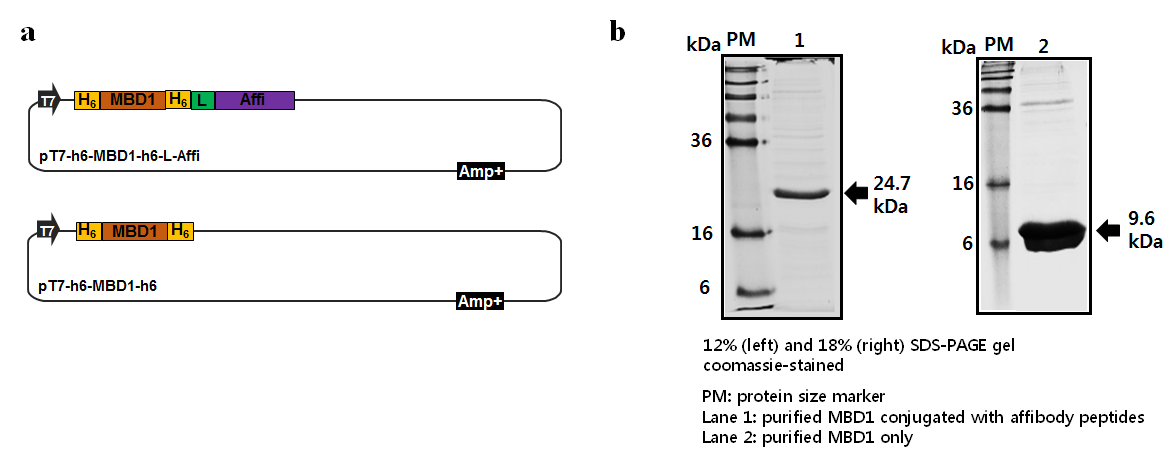
**

**Figure S4. (a)** Plasmid expression vectors used for the synthesis of engineered MBD1 proteins in *E. coli*. "Affibody" represents a peptide with specific and strong affinity for EGFR. "H6" and "L" represent hexahistidine sequence and a flexible linker peptide (G_3_SG_3_TG_3_SG_3_), respectivley. **(b)** SDS PAGE analysis of purified recombinant MBD1 proteins that were synthesized using the plasmid expression vectors, pT7-H6-MBD1-H6-Affi (left gel) and pT7-H6-MBD1-H6 (right gel).

**Figure S5.**

**
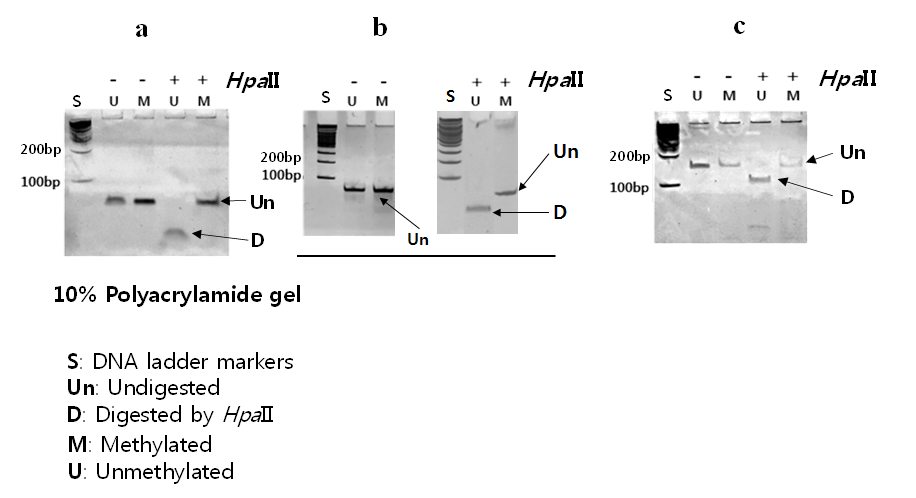
**

**Figure S5**. 10% PAGE images demonstrating the *Hpa*II digestion patterns of the unmethylated and methylated sh-dsDNA backbones: **(a)** 5mCG, **(b)** 9mCG (we used two gels for experiment), and **(c)** 21mCG.

**Figure S6.**

**Figure S6.** Correlations between MBD1 and Au concentrations of DMAs. The Au concentrations were measured by ICP-MS.

**Figure S7.**

**
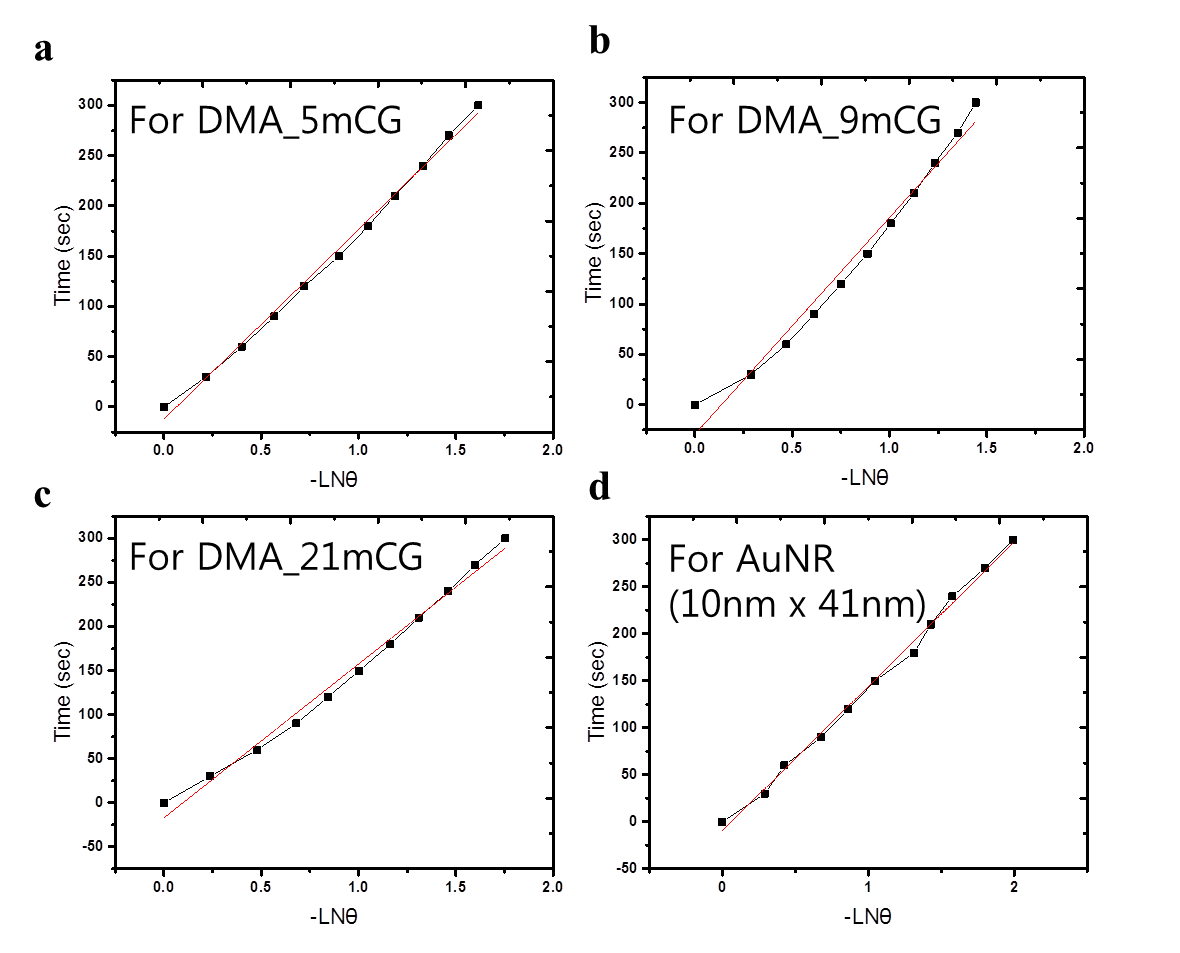
**

**Figure S7.** The heat transfer time constants (τ_s_) were (a) 188.7 sec for DMA_5mCG, (b) 215.2 sec for DMA_9mCG, (c) 174.5 sec for DMA 21_mCG, and (d) 153.5 sec for AuNR, determined by linear regression of the plot, time vs. – ln*θ* (Eqs. 3 and 4 in Experimental Methods) that was obtained from the cooling temperature profiles (Figure 2a). The red lines represent linear fits, and R^2^ values were ~ 0.99 in all cases.

**Supplementary Tables**

**Table S1.** Base sequences of the three sh-dsDNA backbones used in this study. All methylated CG sites are highlighted.

| sh-dsDNA | Sequence (methylation sites in bold; *Hpa*II digestion site (CCGG) underlined) | No. of mCGs | DNA length (bp) |
| --- | --- | --- | --- |
| 5mCG | GTGTAT**C**GCTTAGGTAAT**C**GATTACCTATC**C**GGATAGGTATT**C**GCATACCAATC**C**GTATGG | 5 | 60 |
| 9mCG | GTGTAT**C**GCTTA**C**GTAAT**C**GATTA**C**GTATC**C**GGATA**C**GTATT**C**GCATA**C**GAATC**C**GTATG | 9 | 60 |
| 21mCG | GAGCT**C**GTGTAT**C**GCTTA**C**GTTAT**C**GATTA**C**GTTTC**C**GGATA**C**GTATT**C**GCTTA**C**GAATA**C**GTTTGGCT**C**GAGTCATA**C**GGATT**C**GTATG**C**GAATT**C**GTTTT**C**GAATT**C**GTATT**C**GATTT**C**GTAAG**C**GTAAA**C**GGATCC | 21 | 139 |
